# Speech Registration in Symptomatic Memory Impairment

**DOI:** 10.3389/fnagi.2018.00201

**Published:** 2018-07-09

**Authors:** Salwa Kamourieh, Rodrigo M. Braga, Robert Leech, Amrish Mehta, Richard J. S. Wise

**Affiliations:** ^1^Computational, Cognitive, and Clinical Neuroimaging Laboratory, Division of Brain Sciences, Imperial College London, Hammersmith Hospital, London, United Kingdom; ^2^Center for Brain Science, Harvard University, Cambridge, MA, United States; ^3^Department of Neuroradiology, Charing Cross Hospital, Imperial College Healthcare NHS Trust, Faculty of Medicine, Imperial College London, London, United Kingdom

**Keywords:** memory impairment, speech registration, auditory attention, multiple demand cortex, functional magnetic resonance imaging (fMRI)

## Abstract

**Background**: An inability to recall recent conversations often indicates impaired episodic memory retrieval. It may also reflect a failure of attentive registration of spoken sentences which leads to unsuccessful memory encoding. The hypothesis was that patients complaining of impaired memory would demonstrate impaired function of “multiple demand” (MD) brain regions, whose activation profile generalizes across cognitive domains, during speech registration in naturalistic listening conditions.

**Methods**: Using functional MRI, brain activity was measured in 22 normal participants and 31 patients complaining of memory impairment, 21 of whom had possible or probable Alzheimer’s disease (AD). Participants heard a target speaker, either speaking alone or in the presence of distracting background speech, followed by a question to determine if the target speech had been registered.

**Results**: Patients performed poorly at registering verbal information, which correlated with their scores on a screening test of cognitive impairment. Speech registration was associated with widely distributed activity in both auditory cortex and in MD cortex. Additional regions were most active when the target speech had to be separated from background speech. Activity in midline and lateral frontal MD cortex was reduced in the patients. A central cholinesterase inhibitor to increase brain acetylcholine levels in half the patients was not observed to alter brain activity or improve task performance at a second fMRI scan performed 6–11 weeks later. However, individual performances spontaneously fluctuated between the two scanning sessions, and these performance differences correlated with activity within a right hemisphere fronto-temporal system previously associated with sustained auditory attention.

**Conclusions**: Midline and lateralized frontal regions that are engaged in task-dependent attention to, and registration of, verbal information are potential targets for transcranial brain stimulation to improve speech registration in neurodegenerative conditions.

## Introduction

Impaired memory for recent conversations is a common symptom at the onset of Alzheimer’s disease (AD) but may accompany other neurological or psychiatric conditions. In AD, early pathology affects the medial temporal lobes (Braak and Braak, 1991), but there is a presymptomatic stage prior to clinical presentation at which time pathology will have become widespread throughout the neocortex (Jack et al., 2013; Sepulcre et al., 2017). Therefore, an initial impairment of episodic memory is soon followed by other cognitive deficits, affecting attention and executive functions (Perry and Hodges, 1999). Similar impairments may be present early on in the course of other cortical neurodegenerative conditions, such as dementia with Lewy bodies (Calderon et al., 2001), and in non-neurodegenerative conditions such as depression in older subjects (Pantzar et al., 2014).

Impaired attention will result in poor registration of what a speaker is saying, which in turn could result in unsuccessful later recollection of the conversation. Further, patients complaining of memory impairment will often say that they find it more difficult to participate in conversations at social functions, in contrast to when they converse in a quiet environment. “Top-down” processes, which include selective attention and working memory, are necessary for segregating attended from unattended background speech (Bregman, 1990; Alain and Arnott, 2000; Darwin and Hukin, 2000a, b; Feng and Ratnam, 2000; Brungart, 2001; Conway et al., 2001; Carlyon, 2004; Cusack et al., 2004; Snyder and Alain, 2007; Darwin, 2008; Kerlin et al., 2010; Ding and Simon, 2012; Zion Golumbic et al., 2013; Zhang et al., 2016). Hence, dysfunction of domain-general “top-down” processes during speech registration may be an important determinant of subsequent memory success in clinical populations with memory complaints. Understanding how domain-general brain systems are impaired in patient populations during verbal registration could thus inform therapeutic strategies aimed at improving memory.

This study investigated brain function during the registration of verbal messages in patients presenting with a progressive symptom of poor memory. Thirty-one consecutive patients who were referred with a diagnosis of possible dementia were studied. Diagnostic investigations and further clinical assessment over time suggested that two-thirds of the patients had possible or probable AD. During functional magnetic resonance imaging (fMRI), patients heard sentences presented in the absence or presence of masking background speech. Immediately after each sentence, a yes/no response was required to a question about the attended verbal message, to assess successful attentive listening. The purpose was to investigate whether the systems necessary for attentive listening showed observable changes in activation during speech registration in patients with poor memory. Comparisons were made with data obtained from normal participants (“controls”), reported previously (Kamourieh et al., 2015). This prior study identified three cortical nodes which were sensitive to speech stream segregation during naturalistic, speech-in-speech masked listening conditions; the left planum temporale extending into the inferior parietal lobule, the right anterior insula/frontal operculum (aI/FOp), and the precuneus.

The aI/FOp and inferior parietal lobule in particular have been considered part of a distributed “multiple demand” (MD) system which also includes dorsal anterior cingulate cortex (dACC) and regions of prefrontal cortex near the inferior frontal sulcus (Duncan and Owen, 2000; Duncan, 2010). This system has been proposed to perform domain-general functions such as the sequential mental programming of sub-tasks, monitoring of performance and conflict, or switching between and maintaining task sets—i.e., functions which generalize across multiple types of tasks (MacDonald et al., 2000; Dosenbach et al., 2007, 2008; Duncan, 2010; Fedorenko et al., 2013; Power and Petersen, 2013). The hypothesis of the current study was that patients complaining of poor memory would demonstrate impaired function of the MD system during speech registration. A secondary aim was to administer a central cholinesterase inhibitor, galantamine, to patients over a period of 6–11 weeks to see if there was evidence for improvement in speech registration and/or modulation of task-related activity in MD cortex. Central cholinesterase inhibitors such as donepezil, galantamine or rivastigmine are designed to overcome acetylcholine-deficient brain states, producing temporary symptomatic benefit in some neurodegenerative diseases (Birks, 2006; Bond et al., 2012; Rolinski et al., 2012; O’Brien et al., 2017).

## Materials and Methods

### Participants

The study and the protocol had prior approval from the North West Thames ethics committee. Written informed consent was obtained from all participants in accordance with the Declaration of Helsinki. A total of 53 participants, 22 controls (12 females, 21 right-handed, mean age 66 years, range 51–82), and 31 patients (16 female, 29 right-handed, mean age 73 years, range 59–87), were recruited (see Table 1 for demographics). The mean age difference between the patients and controls was significant (unpaired *t*-test, *P* < 0.01). Patients were recruited on the basis of a prominent symptom of memory impairment, when a diagnosis of a cortical neurodegenerative disease was being considered at the initial assessment. Patients were scanned on two separate sessions which were 6–11 weeks apart. Immediately prior to each session patients were classified based on the following clinical features; the Addenbrooke’s Cognitive Examination-Revised (ACE-R; Mioshi et al., 2006; Larner, 2007); the CANTAB Alzheimer’s Battery^1^, which includes Paired Associate Learning (PAL), a test sensitive to the presence of early dementia (Blackwell et al., 2004; Egerházi et al., 2007); the Geriatric Depression Rating Scale (GDRS; Yesavage et al., 1982); digit span; and the Test for Reception of Grammar (TROG-2^2^) to assess spoken language comprehension. The controls underwent only one scanning session after an assessment on the same screening tests. A diagnostic MRI was performed on all patients, to determine the presence of medial temporal lobe atrophy or other pathology. Progression of cognitive decline at follow-up was also assessed. In a few patients, cerebrospinal fluid examination (total tau to abeta 1–42 amyloid ratio > 1 indicating probable AD) and an amyloid PET scan were available (Dubois et al., 2014). Pure tone audiometry testing (PC Werth Ltd.; Interacoustics Audiometer AS608) was done on 19/22 controls and 30/31 patients prior to both sessions.

The patients were randomly allocated into two groups to assess the effects of galantamine after their first fMRI session. Those who received galantamine (17/31) and those who did not (14/31) had the same mean score on the ACE–R: 80/100, range 56–96 in the untreated group and 50–89 in the treated group. The daily dose of galantamine was increased over 2 weeks from an initial dose to a maintenance dose of 16 mg per day (8 mg twice daily) and continued until the second behavioral assessment and fMRI session 6–11 weeks later.

**Table 1:** Demographics of the patients and controls included in this study.

Patients			
Sex	Age	ACE-R	Comorbidities
M	70	70	nil
F	81	89	nil
M	82	75	Hypertension, hypercholesterolaemia
M	77	78	Hypertension, angina
M	58	83	nil
F	69	79	nil
F	66	76	Hypercholesterolaemia
F	79	96	Hypercholesterolaemia
F	70	65	Hypertension, NIDDM
F	70	90	nil
M	66	94	Hypertension, IHD
F	82	88	Hypertension, hypercholesterolaemia
M	62	64	nil
F	87	76	COPD
F	71	56	COPD
M	85	82	Hypertension, hypercholesterolaemia
M	63	85	nil
M	60	92	nil
F	84	94	nil
F	82	70	nil
M	77	79	nil
M	81	97	nil
M	82	96	nil
F	60	57	nil
F	59	98	nil
M	82	69	Hypothyroidism
M	77	77	nil
F	79	89	nil
M	74	71	nil
F	71	87	nil
F	65	50	Chemotherapy and radiotherapy for breast cancer 6 years prior
**Controls**			
M	63	99	Hypertension, hypercholesterolaemia, IHD
F	64	100	nil
F	67	99	nil
M	65	96	nil
F	67	96	nil
M	51	99	nil
F	60	95	Hypothyroid
F	64	93	nil
F	65	95	nil
M	53	98	nil
M	51	98	nil
M	77	96	IHD
F	82	95	Hypertension
M	64	95	nil
F	62	100	nil
M	62	100	nil
F	73	94	nil
M	71	98	nil
F	68	92	nil
F	79	94	Polymyalgia rheumatica
F	70	91	nil
M	67	95	nil

*M, male; F, female; ACE-R, Addenbrooke cognitive examination—revised; IHD, ischemeic heart disease; COPD, chronic obstructive pulmonary disease; NIDDM, non-insulin dependent diabetes mellitus*.

The patient population was heterogeneous: seven patients were classified as amnestic mild cognitive impairment (MCI) of unknown etiology (three received galantamine), seven as possible AD (four received galantamine), and 14 as probable AD (seven received galantamine). Of the other three patients, all of whom received galantamine, one subsequently developed neurological signs suggestive of corticobasal syndrome, and two were eventually considered to have depression as a cause of their cognitive symptoms. One of these also had a number of cavernomas, and she had a mutation of CCM1.

### Functional MRI Task Design and Image Acquisition

**Figure 1:**
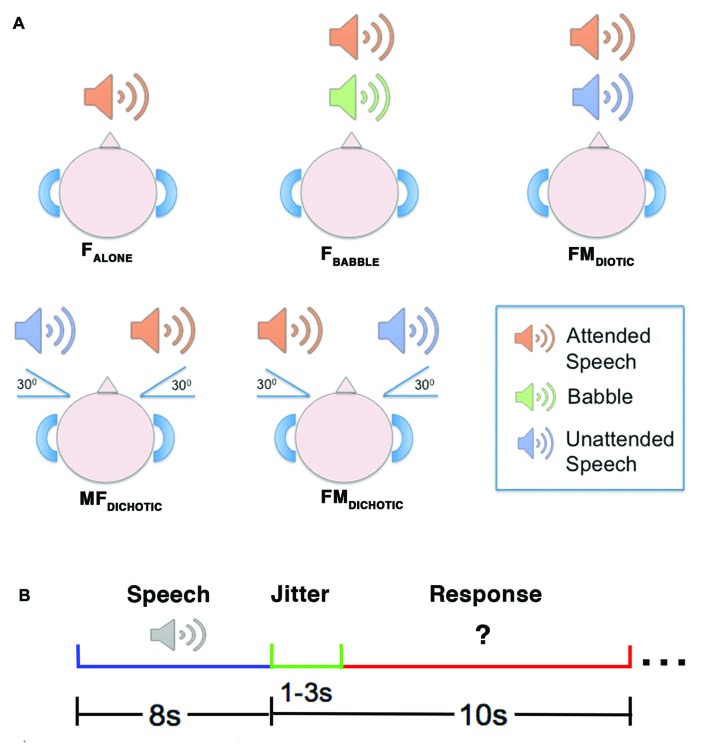
A diagrammatic representation of the auditory conditions heard during the reported study. Upper panel **(A)** depicts the delivery of stimuli. In the F_ALONE_ condition, the attended speaker was presented alone. In F_BABBLE_, background babble (i.e., incomprehensible speech-like sounds) were delivered simultaneously with the attended speaker through the same channel using diotic presentation (i.e., without spatial separation of attended speaker from background). In FM_DIOTIC_, the simultaneous voices of a male and female speaker were presented diotically. In the remaining two conditions, simultaneous female and male speakers were presented dichotically (i.e., with spatial separation of sounds), either with the female speaker at 30° to the left (MF_DICHOTIC_) or vice versa (FM_DICHOTIC_). Lower panel **(B)** depicts the design of an example trial. The speech stimulus was delivered over 8 s, followed by a jitter period (1–3 s) which preceded the written question appearing. The response period then lasted between 7 s and 9 s, depending on the jitter period for that trial. F, Female; M, Male.

The method is the same as that described previously (Kamourieh et al., 2015). During five auditory speech conditions (Figure 1), the participants were required to attend to a female speaker presented through ear-defending MRI-compatible headphones. The female speaker was presented three decibels louder than the background babble or unattended speech in the four masked listening conditions. In three conditions, the female voice originated at 0° in the azimuth plane: speaking alone (F_ALONE_); in the presence of background babble (F_BABBLE_); and in the presence of a male speaker also presented at 0° (FM_DIOTIC_). Two further conditions involved spatial separation, with the male speaker simulated to originate 30° to the right and the female 30° to the left (FM_DICHOTIC_), and vice versa (MF_DICHOTIC_; Algazi et al., 2001). During the scanning sessions, the different listening conditions were presented in a pseudorandomized order over two separate runs of the listening task, ensuring that the number of trials belonging to each condition were evenly distributed across the two runs. Participants were instructed to listen to what the female speaker said and prepare to answer a written question presented on a screen requiring a “yes” or “no” button press response. The questions related to what the female had said in the F_ALONE_ and F_BABBLE_ conditions. In the remaining conditions, that included both male and female speakers, the questions were divided between what the female and the competing male speaker had said.

Interleaved silent steady state (ISSS) imaging was used to ensure that stimuli were heard with minimal background scanner noise (Schwarzbauer et al., 2006). fMRI data acquisition was accomplished using five “imaging” volumes followed by four “quiet” volumes, giving 10 s of scanner acoustic noise followed by 8 s of reduced scanner noise (Kamourieh et al., 2015). Each individual trial consisted of an 8 s listening period (during the “quiet” volumes), followed by a 1–3 s jitter period (to allow sampling of different portions of the hemeodynamic response), followed by a 7–9 s response period (during the acquisition of the five “imaging” volumes; Figure 1B). Imaging volumes were acquired during the response period to allow sampling of delayed hemeodynamic signal changes resulting from the preceding attentive listening periods (Malonek et al., 1997). Each fMRI run contained two periods of fixation lasting 32 s each, which were used as the “REST” baseline.

T2*-weighted gradient echo planar images were collected on a 3T Siemens Tim Trio scanner with a 12-channel phased-array head coil. Thirty-five contiguous axial slices at each of two echo times (13 ms and 31 ms) with a slice thickness of 3 mm were acquired in interleaved order (resolution, 3 × 3 × 3 mm; field of view, 192 × 192 × 105 mm), with a repetition time of 2 s, and 242 volumes were acquired in two runs lasting 14 m 42 s each. To correct for magnetic field inhomogeneities, the manufacturer-provided higher-order shim procedure was used. High-resolution (1 mm isotropic) T1-weighted structural images were also acquired for each subject for image co-registration. Stimuli were presented using the Psychophysics Toolbox (Brainard, 1997) under MATLAB (Mathworks, Natick MA, USA).

### fMRI Data Analysis

fMRI data were analyzed within the framework of the general linear model using FMRI Expert Analysis Tool (FEAT) Version 5.98, part of FMRIB’s Software Library^3^ (FSL). The images were pre-processed with realignment of EPI images for motion correction using MCFLIRT (Jenkinson et al., 2002); non-brain voxels were removed using Brain Extraction Tool (BET, Smith, 2002); spatial smoothing was applied using a 6 mm full-width half-maximum Gaussian kernel, followed by grand-mean intensity normalization of the entire four dimensional dataset by a single multiplicative factor; and finally the data were subjected to high-pass temporal filtering (Gaussian-weighted least-squares straight line fitting, with sigma = 50 s), to correct for baseline drifts in the signal. Time-series statistical analyses were carried out using FMRIB’s Improved Linear Modeling (FILM) with local autocorrelation correction. Registration to high resolution structural and Montreal Neurological Institute standard space images were carried out using FMRIB’s Linear Image Registration Tool (FLIRT).

A fixed effects model was used to combine the two runs at the individual subject level. Individual design matrices were created, modeling the different behavioral conditions. Contrast images of interest in each run and session were produced from these individual analyses and used in the second-level analyses. Final between-subject statistical images were produced using a mixed effects analysis with FMRIB’s Local Analysis of Mixed Effects (FLAME) tool. Correction for multiple comparisons was performed using Gaussian random field-based cluster inference with a height threshold of *Z* > 2.3 and a cluster significance threshold of *P* < 0.05. Individual gray matter density maps, computed from T1-weighted images using the script feat_gm_prepare, available with FSL’s Automated Segmentation Tool (FAST; Zhang et al., 2001), were entered as covariates of no interest, to account for the effects of inter-individual cortical atrophy.

## Results

### Audiometry

A group (controls and patients) × ear (left and right) × frequency (250, 500, 1000, 2000, 4000 Hz) analysis of variance (ANOVA) determined that there was a main effect of group (*F*_(1, 45)_ = 6.9, *P* < 0.05) and frequency (*F*_(4,42)_ = 31.7, *P* < 0.001), but no significant interactions (*F*_(4,42)_ = 2.26, *P* > 0.1). The main effects of frequency and group were the result of greater high tone hearing loss in the patient group. The mean difference in detection threshold was 3.6 dB at 250 Hz and 18.1 dB at 4000 Hz. In neither controls nor patients did within-scanner performance correlate with either the mean hearing threshold across all frequencies or the threshold at 4000 Hz (*P* > 0.1).

### Behavioral Tests and Within-Scanner Performance

The recommended cut-off score of 88/100 on the ACE-R is highly sensitive but less specific for detecting dementia (Mioshi et al., 2006; Larner, 2007). A score >87 is still compatible with a diagnosis of MCI or early dementia. Scores in the controls were all >87 (mean = 96 ± 13). Between-group comparisons were made for scores on the PAL, TROG, GDRS and DS, and the overall accuracy of within-scanner responses (Table 2). The patients with ACE-R scores <88 performed significantly worse than controls across all behavioral tests, including after Bonferroni correction for multiple comparisons. Those with ACE-R scores >87 also differed from the controls by returning a poorer mean score on the PAL, even when the four youngest controls were excluded to achieve age-matched parity between the two groups. Therefore, as a group the patients with ACE-*R* >87 were also impaired compared to the controls on visual memory and new learning. As there was no difference on the PAL between the two patient groups (>87 and <88), the two groups were combined for subsequent analyses.

**Table 2:** Comparison of cognitive test scores.

	ACE-R	In-scanner scores	TROG (total error)	GDS	DS (f)	PAL adjusted (6 shape)
Patients vs. controls	*t* = −5.9, *P* < 0.001 (−22.21, −10.93)	*t* = −6.76, *P* < 0.001 (−27.3, −14.8)	*t* = 2.78, *P* = 0.008 (0.65, 4.06)	*t* = 3.69, *P* = 0.001 (1.07, 3.63)	*t* = −0.81, *P* = 0.42 (−0.98, 0.42)	*t* = 0.002, *P* < 0.001 (10.01, 21.02)
ACE-*R* > 87 vs. controls	*t* = −3.01, *P* = 0.005 (−5.57, −1.07)	*t* = −2.7, *P* = 0.019 (−23.93, −2.54)	*t* = 0.7, *P* = 0.5 (−1.13, 2.22)	*t* = 2.38, *P* = 0.036 (0.16, 4.02)	*t* = −0.21, *P* > 0.5 (−0.97, 0.78)	*t* = 3.87, ***P* = 0.001** (5.57, 18.11)
ACE-*R* < 88 vs. controls	*t* = −10.9, ***P* < 0.001** (−28.34, −19.39)	*t* = −9.64, ***P* < 0.001** (−30.65, −20.02)	*t* = 3.49, ***P* = 0.002** (1.36, 5.34)	*t* = 3.75, ***P* = 0.001** (1.15, 3.83)	*t* = −1.03, *P* = 0.31 (−1.14, 0.37)	*t* = 6.07, ***P* < 0.001** (11.72, 23.47)
ACE-*R* > 87 vs. ACE-*R* < 88	n/a	*t* = 2.63, *P* = 0.014 (2.69, 21.51)	*t* = −2.08, *P* = 0.046 (−5.56, −0.05)	*t* = −0.4, *P* > 0.5 (−2.62, 1.82)	*t* = 0.55, *P* > 0.5 (−0.79, 1.39)	*t* = −1.5, *P* = 0.14 (−13.58, 2.08)

*Independent sample t-tests between patient and control groups for out-of-scanner cognitive screening tests and in-scanner task performance. Results show t value, P value and 95% confidence interval (CI). Bold type represents *P* values < 0.005, after correction for multiple comparisons using Bonferroni. Plain type represents *P* < 0.05, uncorrected. ACE-R, Addenbrooke’s Cognitive Examination—Revised; TROG, Test for Reception of Grammar; GDS, Geriatric Depression Scale; DS (f), digit span forward counting; PAL, Paired Associates Learning (data only available on 19/22 controls). Additional tests not included in the table (reaction time and spatial working memory) from the CANTAB battery were significantly different between groups, but only without correction for multiple comparisons*.

**Figure 2:**
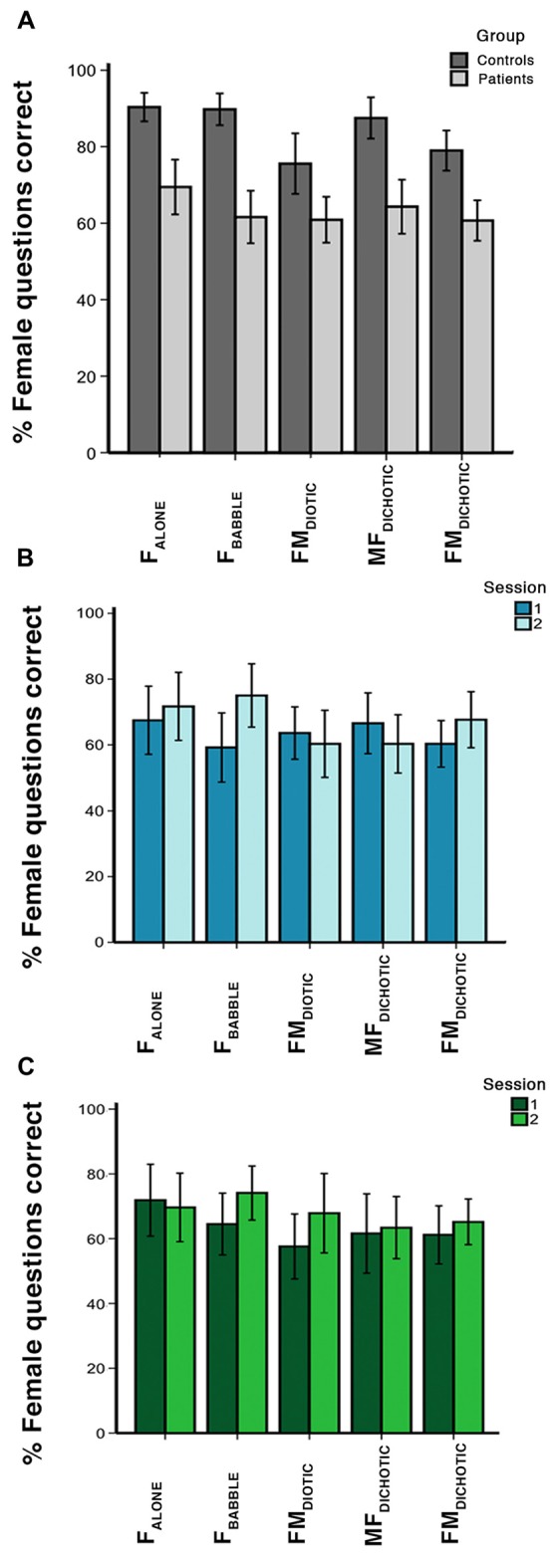
In-scanner behavioral results, showing percentage of questions answered correctly for each auditory condition. Error bars show 95% confidence intervals (CIs). Condition labels are shown in Figure 1. Graphs represent the percentage of questions correct in **(A)** session 1 for the control and patient groups, **(B)** sessions 1 and 2 for the patients who received galantamine between the two sessions, and **(C)** sessions 1 and 2 for the patients who did not receive galantamine between the two sessions.

Within-scanner performance in response to the different listening conditions is summarized in Figure 2. In the controls, a one-way ANOVA demonstrated a difference in performance between in-scanner task conditions (*F*_(4,18)_ = 5.7, *P* < 0.01). Although on paired *t-tests* performance was no different between three conditions (F_ALONE_, F_BABBLE_ and MF_DICHOTIC_; *P* > 0.3, all comparisons), performances during all of these three conditions were better than during F_DIOTIC_ (*P* < 0.05, all comparisons). Performance was better during MF_DICHOTIC_ than during FM_DICHOTIC_ (*P* = 0.02), probably the result of the target speech being presented predominantly to the language-dominant left cerebral hemisphere.

In the patients, a one-way ANOVA also demonstrated a difference in performance between in-scanner task conditions (*F*_(4,27)_ = 3.2, *P* = 0.03). They had a particular problem with the masked speech conditions (F_BABBLE_, FM_DIOTIC_ and FM_DICHOTIC_) compared to the unmasked condition (F_ALONE_; *P* < 0.01, all comparisons), with the exception of MF_DICHOTIC_ (compared with F_ALONE_, *P* = 0.2), when the target speech was presented to the left cerebral hemisphere. In the patients, overall in-scanner task performance across all listening conditions correlated with ACE-R scores at both the first (*r* = 0.5, *P* < 0.05) and second scanning sessions (*r* = 0.6, *P* < 0.001).

Averaging across all listening conditions, the controls and patients (first scanning session only) were significantly better than chance at returning correct responses (*P* < 0.0001 and *P* < 0.001, respectively), but the patients performed poorly compared to the controls across all listening conditions. Repeated measures ANOVA demonstrated a main effect of group (*F*_(1,51)_ = 45.7, *P* < 0.001) and listening condition (*F*_(4,48)_ = 6.6, *P* < 0.001), but no group × condition interaction (*F*_(4,48)_ = 2.03, *P* = 0.11). In summary, the patients had difficulty attending to the target voice in any listening condition.

### Effect of a CChEI on Out-of-Scanner Tests

Behavioral scores from the out-of-scanner behavioral tests performed prior to each fMRI session were not found to show any significant change in either the treated or untreated group. A group (treated and untreated) × session (first and second) × test (eight in total) repeated measures ANOVA showed no effect of group (*F*_(1,29)_ = 0.29, *P* > 0.5) or session (*F*_(1,29)_ = 1.55, *P* > 0.2), and no two- or three-way interactions *P* > 0.1 (test * group *F*_(7,23)_ = 1.16, *P* = 0.36; session * group *F*_(1,29)_ = 0.013, *P* = 0.91; test * session *F*_(7,23)_ = 1.69, *P* = 0.16; test * session * group *F*_(7,23)_ = 1.85, *P* = 0.13). Hence no observable improvement or deterioration in cognitive performance was detected in either the treated or untreated patients.

### Functional MRI Analysis: Controls

**Figure 3:**
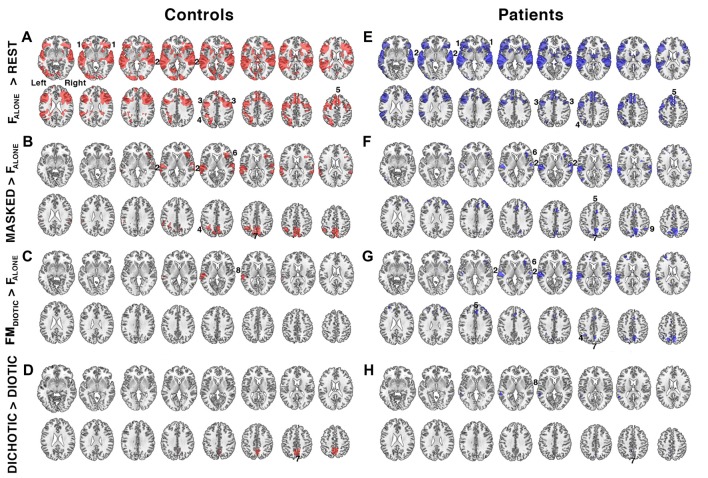
Relative increases in brain activity during different task conditions. **(A–D)** show contrast maps in controls (regions of activity shown in red) and **(E–H)** show the same contrasts in the patient group (in blue). Significant regions of activity were determined using a voxel-level threshold of *Z* > 2.3 and cluster level threshold of *P* < 0.05. Regions included: 1. bilateral anterior insula/frontal operculum (aI/FOp), 2. bilateral auditory cortices, 3. bilateral inferior frontal sulcus, 4. left intraparietal sulcus, 5. dorsal anterior cingulate cortex (dACC), 6. right anterior insula, 7. precuneus, 8. left planum temporale, and 9. right intraparietal sulcus. Contrast maps were produced by comparing the following task conditions (shown in Figure 1): **(A,E)** listening to a single speaker (F_ALONE_) compared to the fixation baseline (REST), **(B,F)** listening to masked speech (MASKED; F_BABBLE_ + FM_DIOTIC_ + MF_DICHOTIC_ + FM_DICHOTIC_) contrasted with listening to a single speaker (F_ALONE_), **(C,G)** listening to non-spatially separated competing speech (FM_DIOTIC_) contrasted with a single speaker (F_ALONE_), **(D,H)** listening to spatially separated (DICHOTIC; MF_DICHOTIC_ + FM_DICHOTIC_) compared to non-spatially separated (DIOTIC; F_BABBLE_ + FM_DIOTIC_) competing speech. Axial slices shown in neurological convention, with right hemisphere on the right side of each slice, beginning 5 mm above the anterior-posterior commissural plane and progressing in 4 mm increments in the Z plane.

Listening to a single speaker (F_ALONE_) contrasted with Rest demonstrated bilateral auditory cortical activation. There was also increased activity in the dACC and superior frontal gyrus and bilateral aI/FOp, the bilateral dorsolateral frontal cortices, just anterior to the precentral sulci, and parietal cortices centered on the intraparietal sulci (activity within the right intraparietal sulcus was only evident at a lower statistical threshold; Figure 3A). These distributed regions overlap with a system that has become known collectively as MD cortex, comprising a cingulo-opercular system (COpS) and a fronto-parietal system (FPS; Dosenbach et al., 2007, 2008; Duncan, 2010; Fedorenko et al., 2013; Power and Petersen, 2013). Whatever the differences in the processes controlled by components of the COpS and the FPS, a subject of active research (Seeley et al., 2007; Hampshire et al., 2012; Aron et al., 2014; Hampshire and Sharp, 2015; Crittenden et al., 2016), most functional neuroimaging studies have observed that both systems become active during many different kinds of task performance.

The second contrast (Figure 3B) was of all masked listening conditions with the single speaker condition (F_BABBLE_ + FM_DIOTIC_ + MF_DICHOTIC_ + FM_DICHOTIC_ > F_ALONE_). This contrast revealed activity associated with speech stream segregation. Increased activity was confined to the right anterior insula, bilateral auditory cortices, the precuneus and the left intraparietal sulcus. A contrast of F_ALONE_ with the attended female speaker masked by the unattended male speaker, but without spatial cues (FM_DIOTIC_ > F_ALONE_), demonstrated significant activity confined to the left posterior auditory cortex, the planum temporale (Figure 3C). A fourth contrast explored the influence of spatial cues by comparing spatial with non-spatial listening conditions (MF_DICHOTIC_ + FM_DICHOTIC_ > F_BABBLE_ + FM_DIOTIC_) and revealed a cluster of activation located within the precuneus (Figure 3D).

### Functional MRI Analyses: Patients

**Figure 4:**
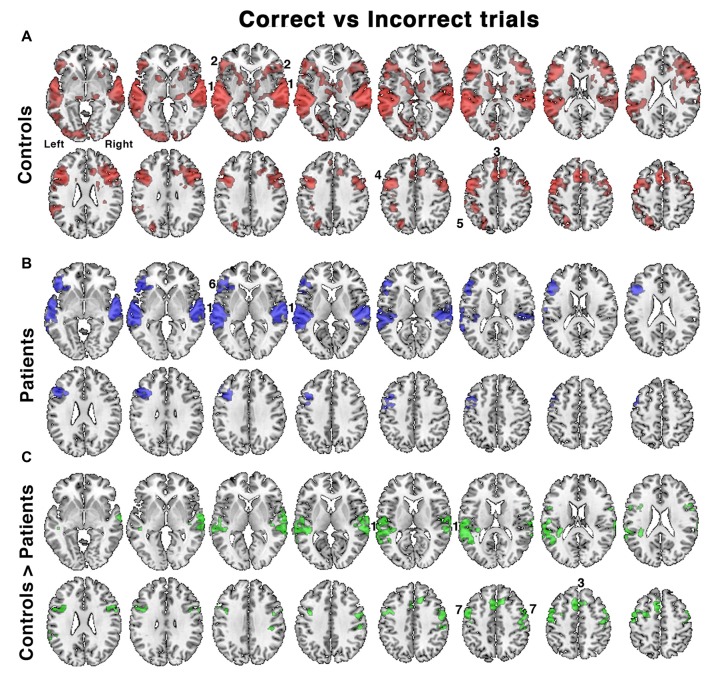
Relative increases in brain activity following successful vs. unsuccessful speech registration trials while listening to all conditions in **(A)** controls and **(B)** patients, as well as **(C)** the comparison of controls > patients. Identified regions included; 1. bilateral auditory cortices, 2. bilateral anterior insula/frontal operculum (aI/FOp), 3. dACC, 4. left inferior frontal sulcus, 5. left intraparietal sulcus, 6. left aI/FOp and 7. bilateral dorsolateral prefrontal cortex. Axial slices displayed as in Figure 3. Significant regions of activity are projected as a red overlay in controls, blue in patients and green for the between-group contrast, with a voxel-level threshold of *Z* > 2.3 and cluster level threshold of *P* < 0.05.

The same four contrasts in the patients demonstrated similar distributions of activity (Figure 4). Where there were apparent differences, these were not significant in a direct comparison between the two groups, even when the individual ACE-R scores were included as a regressor to control for differences in general cognitive ability (theoretically making the analysis more sensitive to task-specific differences in speech registration). Therefore, using these contrasts, and excluding gray matter atrophy and a general measure of cognition as confounds, and taking no account of the difference of in-scanner task performance, the same systems were active in the two groups during attentive listening. Including only the 20 patients with ACE-R scores <88, and therefore more likely to have neurodegenerative pathology, again showed no significant difference from the results observed in the controls.

### Contrasting Listening Trials Followed by Correct and Incorrect Responses

A further analysis investigated the contrast between listening trials followed by a correct response with those followed by an incorrect response. This analysis incorporated differences in performance between the two groups. In the controls accuracy across all listening conditions was ~80%–90%, but only ~60%–70% in the patients (Figure 2). With incorrect responses, there had been impaired processing of the target speech and its representation in working memory, followed by an unlucky guessed response. For correct responses an unknown proportion of responses would have also involved impaired processing but followed by a lucky guess; and this proportion will have been greater in the patient group, especially those with the lowest scores. Hence this contrast (correct > incorrect) was designed to subtract activity associated with guessing, and reveal activity associated with effective processing of the target sentences.

**Figure 5:**
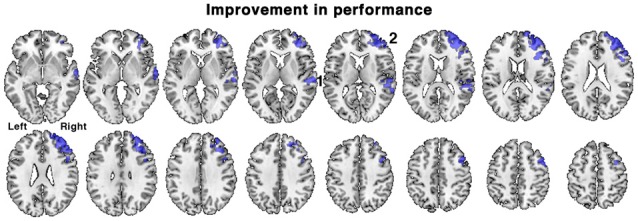
Brain regions associated with an improvement in task performance between the first and second scanning sessions in patients. Regions of activation (shown in blue) demonstrated a positive correlation with percentage change in accuracy of responses during the in-scanner listening task, irrespective of treatment with galantamine. Regions included: 1. right posterior temporal cortex and 2. right dorsolateral prefrontal cortex. Axial slices displayed as in Figure 3. Voxel-level threshold *Z* > 2.3, cluster level threshold *P* < 0.05.

In the controls, there was greater activity in bilateral auditory cortices, the COpS, the left-lateralized FPS and bilateral dorsolateral frontal cortex anterior to the precentral sulci (Figure 4A). In the patients, the distribution of activity appeared more restricted (Figure 4B), and a direct comparison between control and patient groups demonstrated reduced activity in the patients in bilateral superior auditory cortices, including the plana temporale, the dACC/SFG and dorsolateral prefrontal cortices (Figure 4C). Entering the percentage of correct responses as a covariate into the contrast of patients’ correct and incorrect trials, thereby correcting for between-patient individual differences in performance, revealed activity in a distribution almost identical to that illustrated in Figure 5.

### Comparison of CChEI Treatment Groups

Differences in brain activation between the first and second scans in patients with and without CChEI treatment were tested. These group comparisons revealed no overall change in activity between the first and second scans in either treatment group.

### Between-Scan Variability in Task Performance

When all the patients were combined into a single group, the range of percentage change in performance between the first and second scanning sessions was −20.0 to +26.3%, mean = +4.1% ± 11.3, SD). In view of the short interval between the two scans this individual variability in performance likely is not solely attributed to progression of pathology, but perhaps to spontaneous variations in sustained attention over the two scanning sessions (e.g., “good” vs. “bad” days), and other factors such as a training effect. The change in performance was regressed against change in activity between the first and second scanning sessions, using the contrast of all listening conditions followed by correct responses with those followed by incorrect responses, with ACE-R scores included as a regressor of no interest. This analysis revealed what changes in brain activity were correlated with improvements in listening performance, while controlling for differences in the patients’ general cognitive condition and contrasting out activations related to guesses. A positive correlation was observed in right lateralized regions, in right dorsolateral prefrontal cortex centered on the middle frontal gyrus (MFG) and in the right posterior superior temporal sulcus (Figure 5). Similar results were obtained when ACE-R scores were not included as a regressor.

## Discussion

Neurodegenerative and psychiatric conditions are often accompanied by impaired attention and executive functions (Perry and Hodges, 1999; Baddeley et al., 2001; Buckner et al., 2005; Levinoff et al., 2005; Jack et al., 2013; Snyder, 2013; Pantzar et al., 2014). At the time that patients with AD present with memory impairment, high-order neocortices as well as limbic and paralimbic structures are already affected (Buckner et al., 2005; Jack et al., 2013). Further, neuropsychological studies have established that attention is impaired early in the symptomatic course of AD (Perry and Hodges, 1999; Baddeley et al., 2001; Levinoff et al., 2005). This motivated the investigation of impairments in the neural systems responsible for “on-line” attentive listening in patients presenting with a complaint of poor memory, both in the absence and presence of distracting background speech. The study was not directed at a particular neurological or psychiatric disease process, but at what was anticipated might be a common underlying processing impairment in an otherwise heterogeneous group of patients. The hypothesis was that a symptom of forgetfulness for recent conversations may be the consequence of poor attentive registration, either as the sole impairment or one confounding an additional impairment of memory encoding and retrieval.

In both controls and patients, attentive listening with the expectation of a response to a question about the semantic content of the attended verbal message resulted in increased activity throughout bilateral frontal, parietal and temporal regions (Figure 3). This activity was distributed over regions known collectively as MD cortex, which show increased activation during the performance of many different attention-dependent cognitive tasks. MD cortex encompasses two broad systems, the COpS and the FPS (Dosenbach et al., 2007; Duncan, 2010; Menon and Uddin, 2010; Hampshire et al., 2012; Power and Petersen, 2013; Hampshire and Sharp, 2015). In the context of the specific task demands of the present study, increased activity within MD cortex was present irrespective of whether the attended speaker was partially masked by an unattended speaker or not (Figures 3A,E). The data also demonstrated that a number of both auditory and non-auditory regions were involved in the processing of non-spatial and spatial auditory cues that supported the segregation of attended from unattended speech (Figures 3B,F). This was discussed in the earlier article on the results from the controls alone (Kamourieh et al., 2015). A contrast between listening conditions demonstrated the same networks were active in both controls and patients (Figure 3), and this was evident in a sub-analysis including only those patients more likely to have AD.

Further analyses that accounted for individual in-scanner task performance demonstrated that activity in the patients was reduced within both superior temporal cortices and within domain-general midline and lateral frontal cortices in the patients relative to the controls (Figure 4). The distribution of reduced frontal activity closely mimics what has previously been observed as increased activity in normal participants when they attempt to repeat back sentences that have been rendered less intelligible compared to when they had to repeat back clear speech (Brownsett et al., 2014). In a subsequent study, in which patients were studied early and then months after the onset of their post-stroke aphasia, the absolute level of activation within the midline dACC/SFG, a component of the COpS, during both the early and the late scan had a positive predictive value of spontaneous recovery after the stroke (Geranmayeh et al., 2017). It is speculative whether this relationship in any individual patient was due to pre-existing chronic diffuse pathology, such as accumulated hypertensive small vessel disease, or as a result of the acute stroke disrupting the inputs to this region. The present study, on a different patient group, has demonstrated a concordant result, whereby poor in-scanner task performance was associated with reduced activity in the dACC/SFG and aI/FOp (Figure 4), regions that have been identified as part of MD cortex. There was also reduced activity in superior temporal cortex, which included the plana temporale in auditory association cortex posterior to primary auditory cortex. It has been proposed that this region is a “cortical hub” for the processing of auditory information, including the segregation of speech from background noise or unattended speech (Griffiths and Warren, 2002). Whether the reduced activity there was largely due to local neurodegenerative pathology, to reduced “top-down” drive from MD cortex, or both, was not apparent from this study.

**Figure 6:**
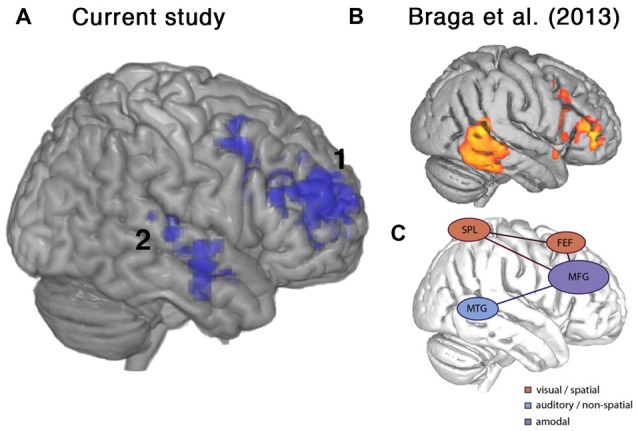
Frontal brain regions associated with improvement on the current listening task overlapped with a putative auditory attention system defined by Braga et al. (2013). **(A)** Lateral view of right hemisphere showing activity from the current study that was positively correlated with percentage change in accuracy, as shown in Figure 5. **(B)** Activations relating to sustained attentive listening observed by Braga et al. (2013) in a study comparing visual and auditory attention in normal participants. **(C)** Schematic figure from Braga et al. (2013) showing proposed top-down attention system containing a domain-general component along the right inferior frontal sulcus and middle frontal gyrus (MFG), which was coupled with modality-specific regions near the frontal eye fields (FEF), superior parietal lobe (SPL) and middle temporal gyrus (MTG) depending on the visual or auditory task demands. Regions shown include: 1. right posterior temporal cortex, 2. right inferior frontal sulcus and MFG.

An unexpected finding was the spontaneous variation in performance between the two scanning sessions, with some participants improving and others worsening between the first and second scans. These changes were unrelated to the degree of cognitive decline in individual patients. Patients that showed improvement in performance also displayed increased activity in right middle and inferior frontal gyri and right lateral temporal cortex (Figures 5, 6A). In the frontal lobe, the anatomical distribution of this system overlapped with putative MD regions in the MFG, just anterior to the precentral sulcus, hypothesized to subserve domain-general functions (Duncan and Owen, 2000; Duncan, 2010) and sustaining attention (Coulthard et al., 2006; Singh-Curry and Husain, 2009). Further, task-improvement-related activations extended along the right inferior frontal sulcus and into pars triangularis, overlapping with a putative auditory attention system that shows increased activity during attentive listening (Figure 6; Braga et al., 2013; Michalka et al., 2015). Thus, rather than being associated with left-hemisphere frontotemporal regions necessary for speech production and comprehension, improvements in the present language task were accompanied by increased activity in a right hemisphere frontotemporal system that likely subserves multiple functions including focusing and maintaining attention to auditory stimuli.

No changes in cognitive measures, in-scanner performance or task-related fMRI activity were observed after administration of a central cholinesterase inhibitor, galantamine, for 6–11 weeks. This may reflect the relatively small and heterogeneous population of patients. A relatively small proportion of patients with AD respond to a CChEI (Kaduszkiewicz et al., 2005). It is possible that the efficacy of CChEI treatment may be a consequence of the pattern and extent of pathology within right hemisphere systems such as those identified here. Namely, drug treatment may prove beneficial when pathology is not so advanced as to preclude a response to increasing central acetylcholine levels in domain-general frontal regions (Nobili et al., 2002). Alternative modulatory neurotransmitter agonists may also prove more effective (Bentley et al., 2008; Gorgoraptis et al., 2012). Although the current patient group was heterogeneous, we focused on the imaging changes associated with a specific symptom (memory impairment) and its modulation with a cholinesterase inhibitor. Further work will be required to delineate whether individual disease processes will dissociate at a network level even if presenting with identical symptoms. Furthermore, extensive analysis in a much larger group will be necessary to determine the effects of co-morbidities (as in Table 1).

Acetylcholine is involved in the regulation of attentional systems (Klinkenberg et al., 2011), but modulatory monoaminergic neurotransmitter systems are also deficient in AD (Šimic et al., 2017), and are dysfunctional in psychiatric disorders (Hamon and Blier, 2013; Di Giovanni et al., 2016), and they influence many frontal executive functions (Robbins and Roberts, 2007). Using a cocktail of agonists may be one approach to improve attentive registration during conversation, but one at risk of causing unacceptable side effects, particularly in the elderly. An alternative is transcranial stimulation to accessible cortical sites. However, a major uncertainty is at which cortical site to apply the stimulation. A search through ClinicalTrials.gov reveals that many groups are investigating transcranial stimulation to improve symptoms in both neurodegenerative and psychiatric conditions, with proposals that cover many different sites. We would expect that the clinical implications from the present study, that by increasing activity in either midline dACC/SFG, to improve selective attention, or to right frontal cortex, to even out longer-term fluctuations in sustained attention, might be targets for future trials of symptomatic treatment of complaints of “forgetfulness.” Further, deep brain stimulation treatments, such as those designed specifically to directly improve memory recollection (Hescham et al., 2013; Lozano et al., 2016), might instead be applied to domain-general systems to improve the registration of verbal information.

## Author Contributions

SK, RL and RW conceived the study. SK and RW recruited the normal participants and the patients. SK, RB and RL designed and performed the analyses. SK wrote the first draft of the manuscript. All authors were involved in drafting subsequent versions through to a completed manuscript.

## Conflict of Interest Statement

The authors declare that the research was conducted in the absence of any commercial or financial relationships that could be construed as a potential conflict of interest.

## Footnotes

^1^www.cambridgecognition.com

^2^www.pearsonclinical.co.uk

^3^www.fmrib.ox.ac.uk/fsl
